# Structural basis for ligand recognition and signaling of the lysophosphatidylserine receptors GPR34 and GPR174

**DOI:** 10.1371/journal.pbio.3002387

**Published:** 2023-12-04

**Authors:** Guibing Liu, Xiu Li, Yujing Wang, Xuan Zhang, Weimin Gong

**Affiliations:** 1 Division of Life Sciences and Medicine, University of Science and Technology of China, Hefei, People’s Republic of China; 2 Department of Pharmacology and Chemical Biology, University of Pittsburgh School of Medicine, University of Pittsburgh, Pittsburgh, Pennsylvania, United States of America; University of Zurich, SWITZERLAND

## Abstract

Lysophosphatidylserine (LysoPS) is a naturally occurring lipid mediator involved in various physiological and pathological processes especially those related to the immune system. GPR34, GPR174, and P2Y10 have been identified as the receptors for LysoPS, and its analogues have been developed as agonists or antagonists for these receptors. However, the lack of structural information hinders the drug development with novel characteristics, such as nonlipid ligands and allosteric modulators. Here, we determined the structures of human GPR34 and GPR174 in complex with LysoPS and G protein by cryo-EM. Combined with structural analysis and functional studies, we elucidated the lipid-binding modes of these receptors. By structural comparison, we identified the structural features of GPR34 and GPR174 in active state. Taken together, our findings provide insights into ligand recognition and signaling of LysoPS receptors and will facilitate the development of novel therapeutics for related inflammatory diseases and autoimmune diseases.

## Abstract 2

Lysophosphatidylserine (LysoPS) is a lipid signalling molecule implicated in a range of processes related to the immune system. This structural study reveals the mechanism of LysoPS recognition by two G protein-coupled receptors, GPR34 and GPR174, offering insights into lysophospholipid signaling and presenting opportunities for drug discovery.

## Introduction

Lysophospholipids (LPLs) are single-chain lipid mediators generated by phospholipid metabolism and regulate various physiological processes including cell survival, proliferation, and migration and play a role in many diseases of vascular, neurological, and metabolic systems [1,2]. Lysophosphatidic acid (LPA), sphingosine 1-phosphate (S1P), lysophosphatidylserine (LysoPS), and lysophosphatidylcholine (LPC) represent the most widely studied LPLs (S1A Fig). Of these, LysoPS has been shown to be important in cell migration [3,4], cell differentiation [5], and oxidative stress [6]. Additionally, LysoPS has been reported to induce mast cell degranulation [7,8] and promote the phagocytosis of apoptotic cells [9]. In human, 3 G protein–coupled receptors have been identified as LysoPS receptors, namely, GPR34, GPR174, and P2Y10. The first LysoPS receptor GPR34 was discovered through the observation of LysoPS-induced inhibition of cAMP accumulation in GPR34-expressing CHO cells [8]. Subsequently, GPR174 and P2Y10 were found to activate Gα_12/13_ in response to LysoPS, as demonstrated by the transforming growth factor-α (TGFα) shedding assay [10].

GPR34 is a Gi-coupled LysoPS receptor that exhibits high expression in microglia, mast cells, lymphocytes, and macrophages [11] and is associated with immune and neurological disorders (ref). In addition to its involvement in delayed hypersensitivity [12] and dendritic cell apoptosis [13], GPR34 is also implicated in tissue repair, as it senses LysoPS released by apoptotic neutrophils on type 3 innate lymphoid cells [14]. Activation of GPR34 has been linked to neuropathic pain [15] and the development of salivary gland MALT lymphoma [16], indicating its potential as a therapeutic target in these conditions. GPR174 is highly expressed in lymph nodes and thymus and has been associated with autoimmune diseases such as Graves’ disease [17] and Addison’s disease [18]. GPR174 negatively regulates regulatory T cell (Treg) proliferation and function through a Gα_s_-dependent pathway in response to LysoPS and inhibits CD4 T cell proliferation and activation by reducing IL-2 production. A recent study also provides evidence for the role of GPR174 in regulating gene expression and function of B cells via Gα_s_ [19]. In addition, GPR174 was found to induce B cell migration in response to CCL21, which promotes GPR174-Gα_i_ coupling in a sex-dependent manner [20]. However, the role of Gα_12/13_ pathway in GPR174 signaling is poorly understood, and the coupling of Gα_i_ to GPR174 is relatively limited [21]. The Gα_12/13_-coupled P2Y10 expressed in immune cells has not been extensively studied. Specifically, P2Y10 has been reported to mediate eosinophils degranulation [22] and facilitate chemokine-induced CD4 T cell migration [23]. Notably, P2Y10 also responds to ATP as a purinergic receptor [23] and may also respond to S1P [24], which distinguishes it from GRP34 and GPR174.

GPR174 primarily couples to the stimulatory G protein Gs, whereas GPR34 exclusively couples to the inhibitory G protein Gi/o [21,25], both of which exert their effects by regulation of cAMP level in cells. However, the basis underlying G protein selectivity is not understood. Furthermore, GPR174 shares a high homology (up to 48.2%) with the putative P2Y receptor P2Y10, but a relatively low homology (28.5%) with GPR34, which was originally classified as a P2Y-like orphan receptor. It is unclear whether GPR34 and GPR174 share the same recognition mechanism for LysoPS, which hinders rational drug design for LysoPS receptors. Therefore, we used cryo-EM to determine the structures of GPR34 and GPR174 in complex with LysoPS and their appropriated G proteins. In combination with mutagenesis and functional assays, these structures reveal the basis for ligand recognition and G protein coupling of LysoPS receptors. Our study provides valuable insights for structure-based drug design targeting LysoPS receptors.

## Results

### Structures of LysoPS-signaling complexes

**Fig 1:**
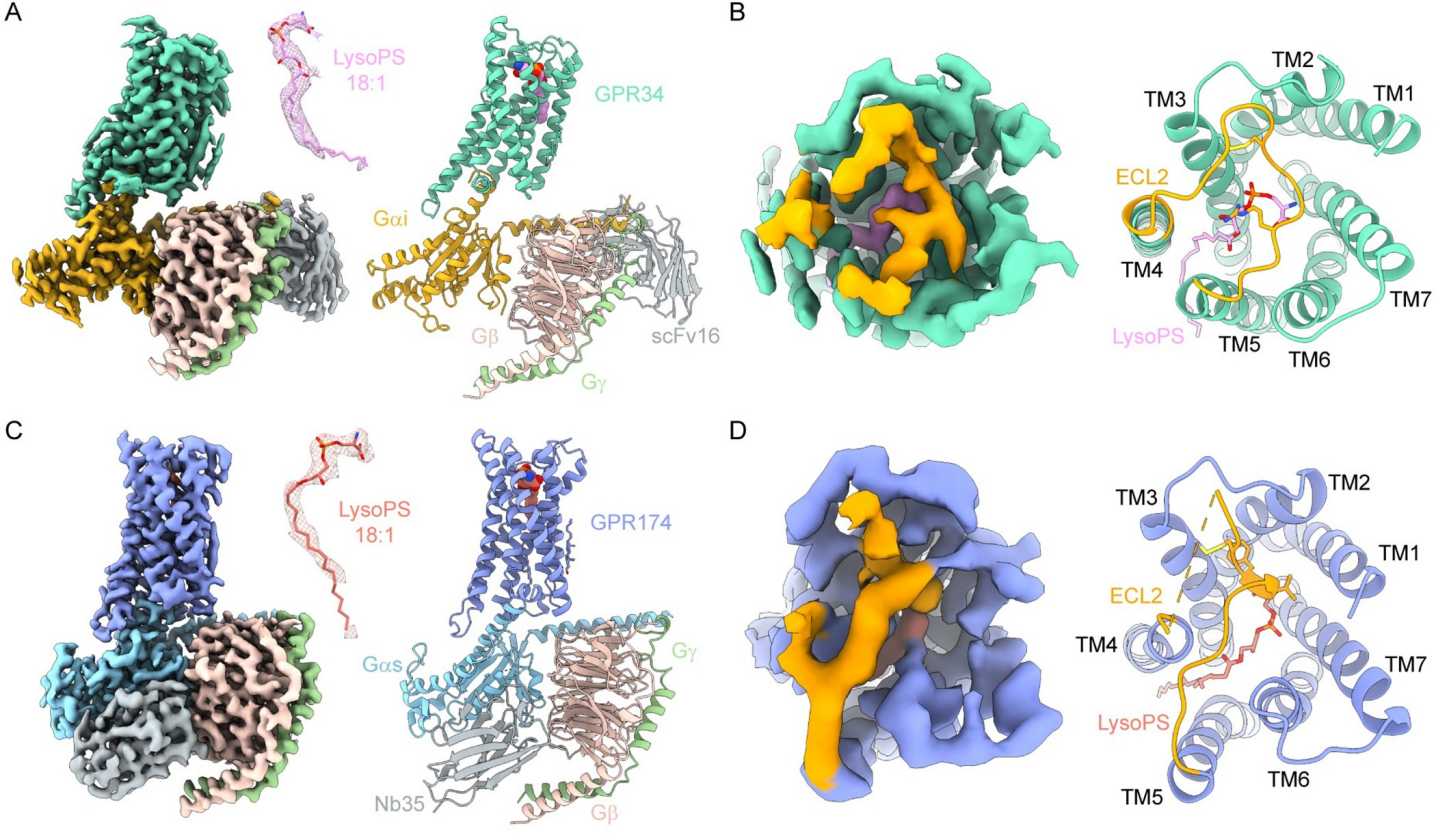
Overall structures of GPR34-Gi complex and GPR174-Gs complex. (**A**) Cryo-EM density map and model of GPR34-Gi complex. Density of LysoPS is shown as mesh in the middle and colored plum. (**B**) The map and model of ECL2 of GPR34-Gi complex (extracellular view). (**C**) Cryo-EM density map and model of GPR174-Gs complex. Density of LysoPS is shown as mesh in the middle and colored salmon. (**D**) The map and model of ECL2 of GPR174-Gs complex (extracellular view). GPR34 is colored marine green. GPR174 is colored blue. ECL2 is colored orange. Gα_i_, Gα_s_, Gβ, and Gγ subunits are colored gold, cyan, pink, and light green, respectively. ScFv16 and Nb35 are colored grey.

We coexpressed the GPR34-Gi and GPR174-Gs signaling complexes in Sf9 insect cells and purified them in the presence of LysoPS (18:1), one the most widely used LysoPS in functional studies of LysoPS receptors. ScFv16 and Nb35 were incorporated to stabilize the Gi and Gs complex, respectively. The NanoBiT tethering strategy [26] was also utilized to facilitate the formation of the complexes and improve the quality of cryo-EM samples. As a result, we successfully acquired high-quality structures of LysoPS-bound GPR34-Gi complex and LysoPS-bound GPR174-Gs complex at resolution of 2.91 Å and 3.06 Å, respectively (Figs 1A, 1C, S2 and S3 and S1 Table). The well-defined density allowed for precise modeling of GPR34-Gi, GPR174-Gs complexes, and the binding ligands. In both structures, we modeled LysoPS (18:1) into the density and found that it fits well except that the last 6 carbons of the oleic acid have no density.

Notably, the lipid-binding pocket of both GPR34 and GPR174 is covered by a lid formed by ECL2, which is stabilized by the conserved disulfide bond with TM3 (Fig 1B and 1D). Additionally, ECL2 also serves as part of the lipid-binding pocket, as seen in other lipid receptors.

### Binding modes of LysoPS to GPR34 and GPR174

**Fig 2:**
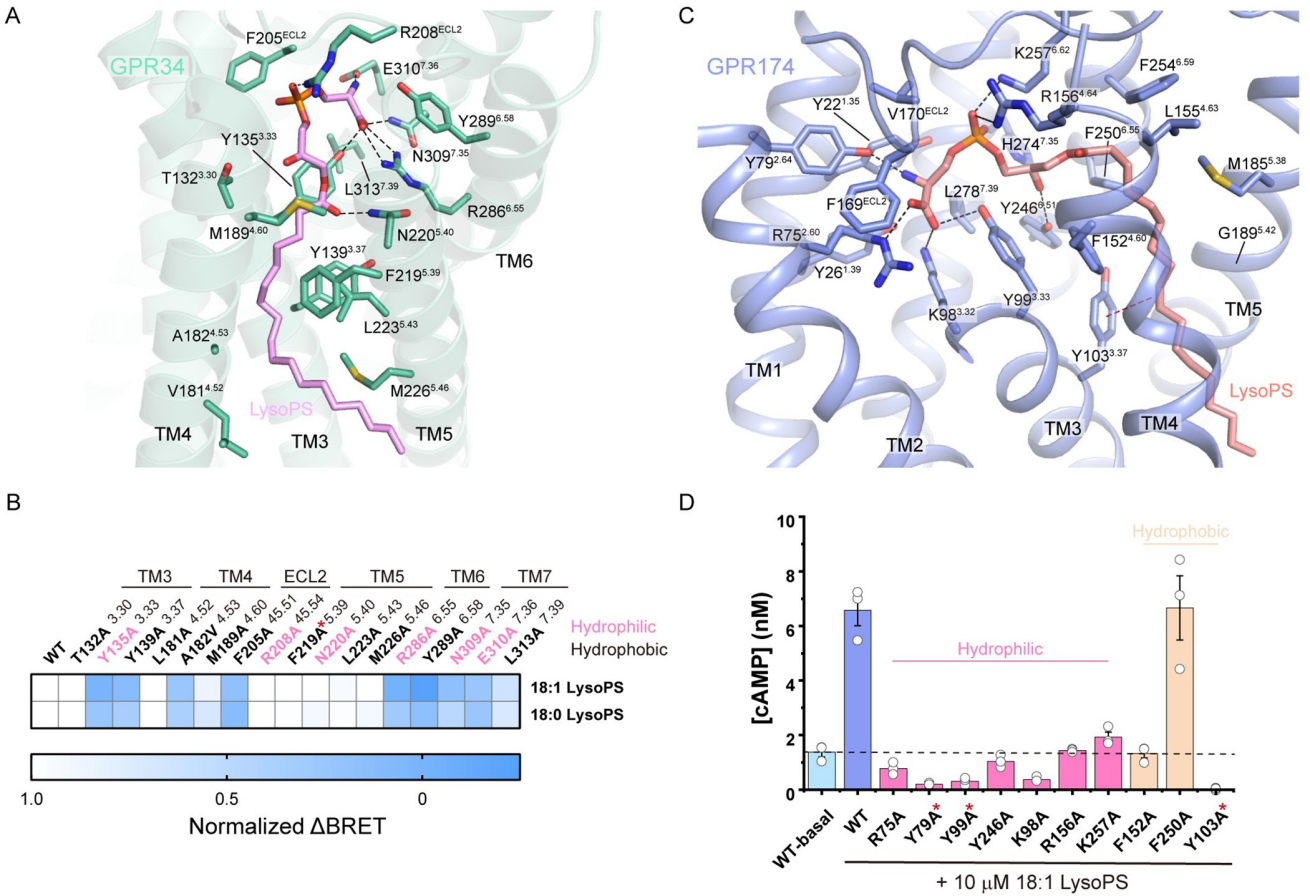
Recognition of LysoPS by GPR34 and GPR174. (**A**) Interactions between LysoPS and GPR34. Polar interactions are depicted as black dashed lines. (**B**) Normalized ΔBRET of GPR34 mutants in response to 10 μM LysoPS relative to wild-type GPR34 in Gi-dissociation assays. (**C**) Interactions between LysoPS and GPR174. Polar interactions are depicted as black dashed lines. The π–π interaction is depicted as a red dashed line. (**D**) Net cAMP accumulation of CHO cells expressing GPR174 mutants in response to 10 μM LysoPS. WT-basal represents net cAMP accumulation of cells expressing wild-type GPR174 in the absence of exogenous LysoPS. Data represent mean ± SEM from 3 independent experiments. Mutants with expression less than half that of the wild-type receptor are labeled with red stars. The data used to generate graphs in Fig 2B and 2D are available in S1 Data.

LysoPS binds to GPR34 by a specific interaction network between the ligand and the receptor’s transmembrane domain (TMD) and ECL2 (Fig 2A). The polar head group of LysoPS is accommodated in the central cavity of the TMD, where it forms hydrogen bonds and salt bridges with several key residues. Specifically, the L-serine moiety of LysoPS interacts with TM6 and TM7, with the carboxyl group forming a salt bridge with R286^6.55^ and hydrogen bonds with Y135^3.33^ and N309^7.35^, while the amino group forms a salt bridge with E310^7.36^. The phosphate group of LysoPS interacts with R208^ECL2^ via a salt bridge. Additionally, the ester linkage between the fatty acid and the polar head forms a hydrogen bond with N220^5.40^. The formation of the central pocket also involves hydrophobic interactions with several other residues, including T132^3.30^, Y289^6.58^, F205^ECL2^, and L313^7.39^. The acyl tail of LysoPS is bound in a hydrophobic subpocket formed by the sidechains of Y139^3.37^, L181^4.52^, A182^4.53^, M189^4.60^, F219^5.39^, L223^5.43^, and M226^5.46^. Mutagenesis studies using a Gi-dissociation assay in HEK293T cells with both 18:1 and 18:0 LysoPS (Figs 2B and S4) revealed that several residues in contact with the polar head of LysoPS are critical for LysoPS-induced activation of GPR34, including Y135^3.33^, F205^ECL2^, R286^6.55^, Y289^6.58^, E309^7.35^, and E310^7.36^, which severely impaired receptor activation when mutated to alanine (S4C and S4D Fig). L313^7.39^ mutation moderately affected receptor activity. In contrast, R208^ECL2^ mutation has little effect on receptor activation, suggesting that this site may not be crucial for LysoPS-induced activation and could potentially be substituted by surrounding positively charged residues such as K128^3.26^, H199^ECL2^, H206^ECL2^, and K210^ECL2^ in ligand binding. Mutations of most residues in the hydrophobic subpocket have no effect on receptor activation, except for Y139^3.37^A, A182^4.53^V, and M189^4.60^A (S4E Fig). These findings indicate that the recognition of LysoPS by GPR34 is primarily mediated by specific interactions with the polar head group, while the hydrophobic interactions with the acyl tail provide additional stability to the binding.

Previous evolutionary studies found that GPR34 receptors have existed for more than 450 million years and identified residues conserved during GPR34 evolution [27,28]. Of the residues involved in LysoPS recognition, Y135^3.33^, Y139^3.37^, R208^ECL2^, N220^5.40^, R286^6.55^, Y289^6.58^, N309^7.35^, E310^7.36^, and L313^7.39^ of GPR34 are highly conserved during vertebrate evolution. Notably, all 6 residues associated with LysoPS by polar interactions are conserved. Most mutations of these residues impaired GPR34 activation except R208^ECL2^ and N220^5.40^ (Fig 2B).

GPR174 binds the polar head of LysoPS in a central pocket through extensive polar interactions (Fig 2C). Specifically, the carboxyl group of the serine moiety forms salt bridges with R75^2.60^ and K98^3.32^, and a hydrogen bond with Y99^3.33^, while the amino group forms a hydrogen bond with Y79^2.64^. Additionally, the phosphate group forms ionic interactions with R156^4.64^ and K257^6.62^, and the *sn-2* hydroxyl group contacts Y246^6.51^ by a hydrogen bond. F169 and V170 of ECL2 participate in the formation of the central pocket by hydrophobic interactions, with F169 further stabilizing the binding of the polar head through a cation–π interaction with R75^2.60^. The acyl tail of LysoPS binds in a narrow cleft formed by hydrophobic residues from transmembrane helices 3 to 6, including Y103^3.37^, F152^4.60^, M185^5.38^, and F250^6.55^, with the double bond of the oleoyl group in contact with Y103^3.37^ through a π–π interaction. G189^5.42^ and G193^5.46^ on TM5 provide space for the binding of the acyl group. cAMP accumulation assays were performed in CHO cells to assess the contributions of the residues involved in LysoPS binding (Figs 2D and S5). Mutations to alanine at the polar interaction sites substantially impaired or even abolished LysoPS-induced cAMP accumulation, while mutations of hydrophobic sites like F152A and Y103A have similar effects.

**Fig 3:**
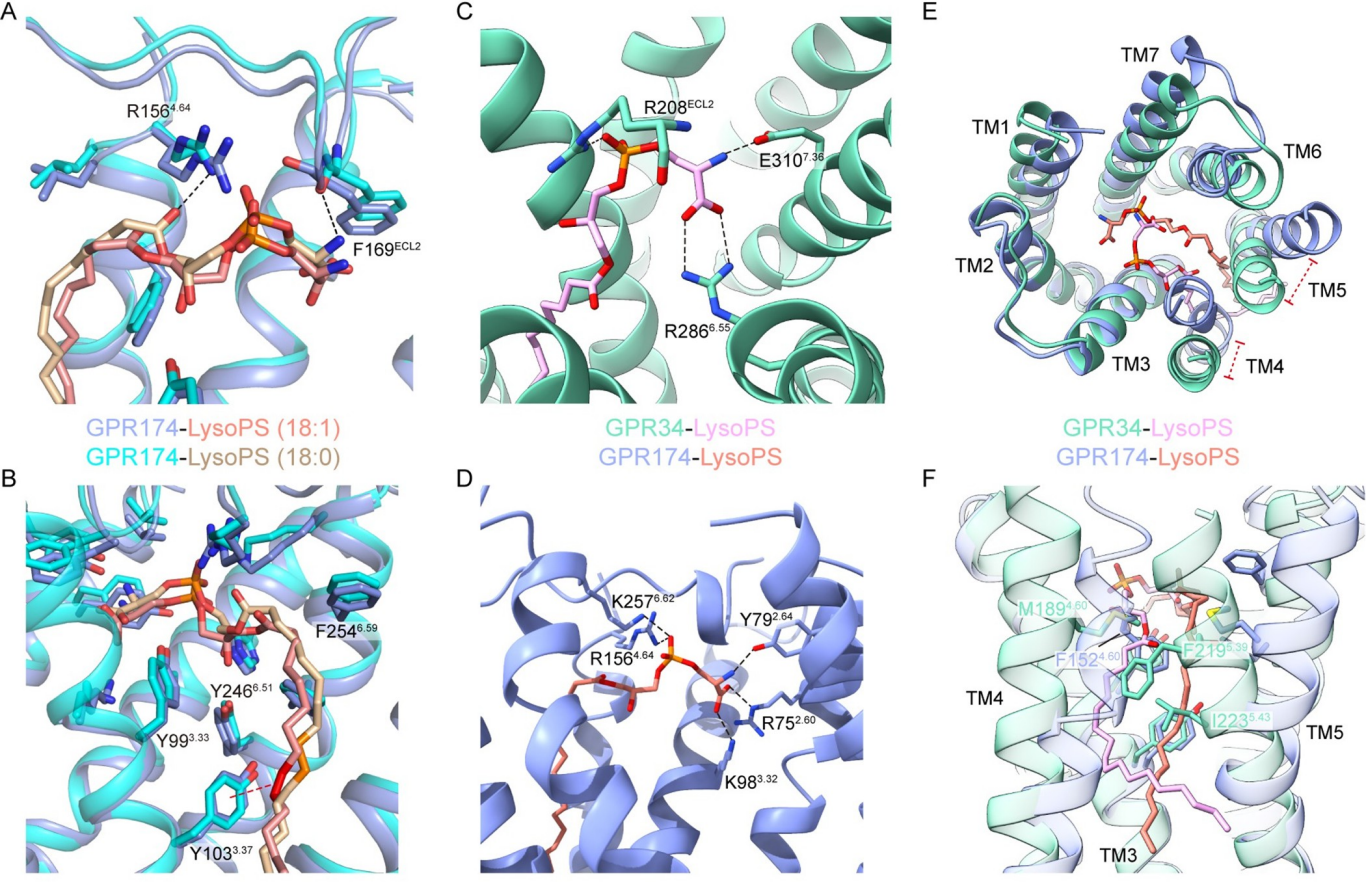
Comparison of LysoPS recognition by GPR34 and GPR174. (**A**) Interactions between GPR174 and the polar head of LysoPS (18:0) or LysoPS (18:1). (**B**) Interactions between GPR174 and the acyl tail of LysoPS (18:0) or LysoPS (18:1). The double bond of LysoPS (18:1) is colored red; the corresponding single bond in LysoPS (18:0) is colored orange. (**C**) Charged interactions between LysoPS and GPR34 in the positively charged pocket. (**D**) Charged interactions between LysoPS and GPR174 in the positively charged pocket. (**E**) Structural superposition of LysoPS bound GPR34 and GPR174 (extracellular view). (**F**) Comparison between acyl tails of LysoPS binding in GPR34 and GPR174. Polar or charged interactions are depicted as black dashed lines.

We noticed the recently reported structure of the LysoPS (18:0)-GPR174-Gs complex [29] and compared it with our LysoPS (18:1)-bound complex. Interestingly, the same polar head of the 2 types of LysoPS binds to the central pocket with some unexpected differences (Fig 3A). In our structure, the carboxyl group of the serine moiety does not form a hydrogen bond with the main chain carbonyl of F169^ECL2^, and the hydrogen bond between the carbonyl group of the ester linkage and R156^4.64^ is also absent. Furthermore, we observed an additional π–π interaction between the double bond on the acyl group of LysoPS (18:1) and the phenol ring of Y103, which may result in tighter binding (Fig 3B).

The interaction patterns of LysoPS with GPR34 and GPR174 are different. In both receptors, the polar head binds in a positively charged central pocket (Fig 3C and 3D), but in contacts with different sets of residues. In GPR34, the serine carboxyl and phosphate group of LysoPS form ionic interactions with R286^6.55^ and R208^ECL2^, respectively (Fig 3C), while in GPR174, their orientations are determined by K98^3.32^, R75^2.60^, R156^4.64^, and K257^6.62^ (Fig 3D). In GPR34, amino group of the serine in LysoPS forms a salt bridge with E310^7.38^, whereas in GPR174, it forms a hydrogen bond with Y79^2.64^. The polar head of LysoPS makes more charged interactions with GPR174, suggesting a tighter binding than with GPR34. Regarding the hydrophobic tail, its orientation is mainly determined by the arrangement of TM4 and TM5 (Fig 3E and 3F), which are differentiated by prolines on them. In GPR34, there are no prolines on TM4/5, including the conserved P^5.50^ among class A GPCRs. A curved path extends against TM3 and TM4, further restricted by L223^5.43^. In GPR174, P153^4.61^ and P197^5.50^ result in a distinct arrangement of TM4 and TM5, forming a straight path with hydrophobic residues from TM3-6. L^5.43^ is replaced by a glycine at position 5.42 in GPR174.

Overall, our findings suggest that while GPR34 and GPR174 share similar binding modes for LysoPS, they exhibit differences in the specific binding residues and the hydrophobic tail’s orientation.

### Lipid-binding modes of lysophospholipid receptors

**Fig 4:**
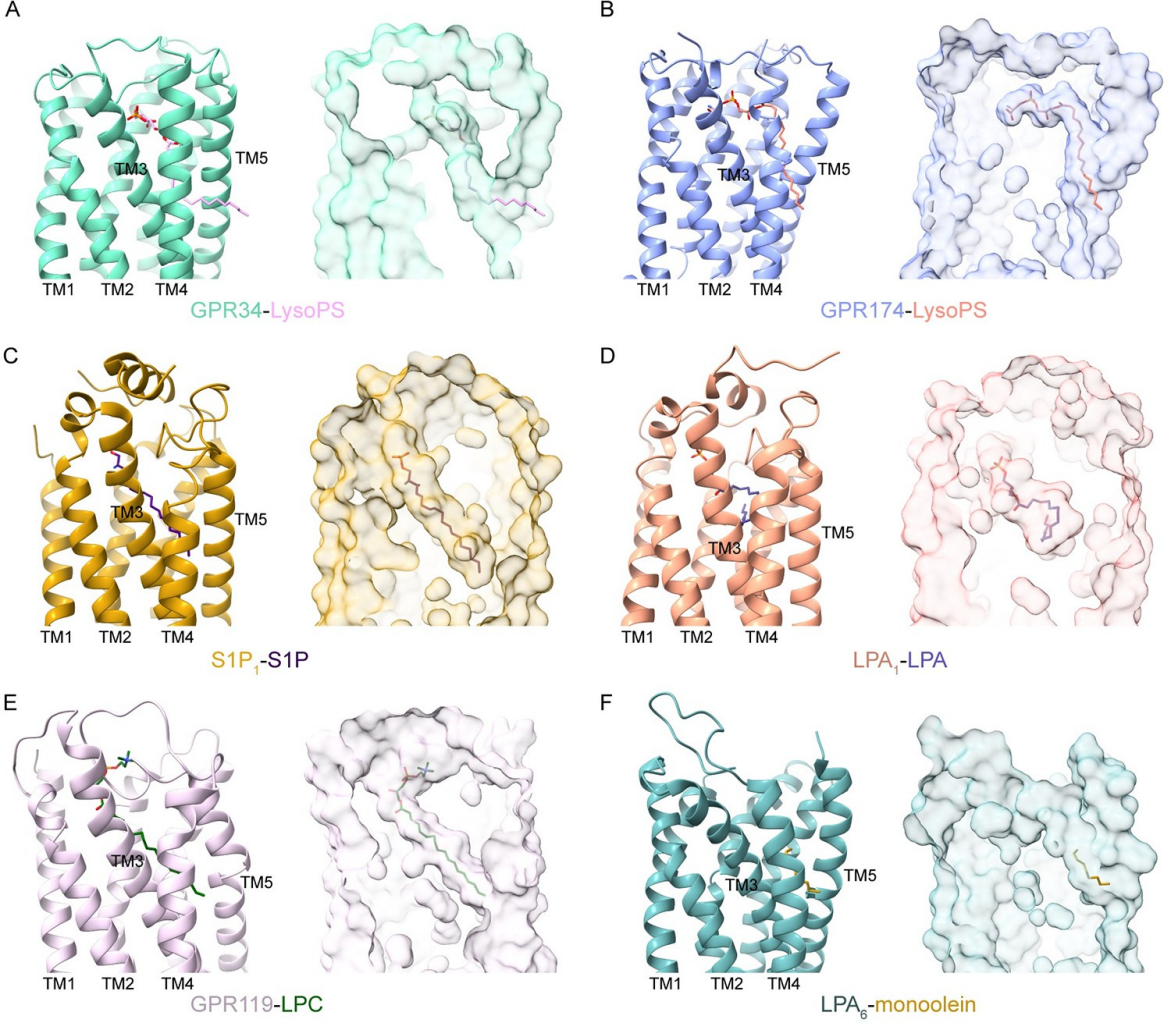
Comparison of lysophospholipid-binding modes. The ligand-binding pockets of GPR34 (**A**), GPR174 (**B**), S1P_1_ (**C**, PDB: 7td3), LPA_1_ (**D**, PDB: 7dt0), GPR119 (**E**, PDB: 7xz5), and LPA_6_ (**F**, PDB: 5xsz). GPR34, GPR174, S1P_1_, LPA_1_, GPR119, and LPA_6_ are colored marine green, blue, dark gold, orange, plum, and light blue, respectively. The ligands are shown as sticks. The blobs represent the surface of the atomic models.

The binding modes of several other LPLs have been illustrated by previous structural studies, including LPA receptors [30–32], S1P receptors [33–39], and the LPC receptor GPR119 [40]. S1P receptors (S1P_1-5_) are known as the endothelial differentiation gene (EDG) family. LPA_1-3_ also belong to the EDG family, while LPA_4-6_ belong to the P2Y family (S1B Fig). Meanwhile, GPR34 and GPR174 are considered P2Y-like orphan receptors [1]. Notably, the binding pocket of LysoPS in GPR34 or GPR174 is distinct from that of S1P, LPA, and LPC in their respective receptors. Specifically, in the structure of GPR34 and GPR174, the polar head of LysoPS is enveloped by the 7-TM bundles, while the hydrophobic tail extends to the cleft formed by TM3-5 (Fig 4A and 4B), thereby creating a semi-open pocket for LysoPS. Conversely, in the structures of endogenous ligand-bound S1P_1_, LPA_1_, and GPR119, the ligand-binding pockets are almost occluded, wherein S1P, LPA, and LPC are surrounded by TM helices (Fig 4C–4E). Interestingly, in the crystal structure of LPA_6_, one of the monoolein molecules, although incomplete, binds in a pocket similar to that of LysoPS in GPR34 and GPR174 (Fig 4F). Additionally, the platelet activating factor receptor (PAFR), another P2Y-like receptor, is highly similar in sequence to GPR34. Docking analysis revealed that PAFR employs a similar lipid-binding mode to that of GPR34 and GPR174 [41]. By comparing the ligand-binding modes of different LPL receptors, we suggest that the semi-open lipid-binding pocket may be shared by other P2Y-like lipid receptors including LPA_4-6_ and PAFR.

### G protein coupling

GPR34 and GPR174 modulate cAMP level in cells via Gi and Gs pathways, respectively. We analyzed the G protein–coupling profiles of these 2 receptors to gain insights into the basis of their G protein selectivity.

**Fig 5:**
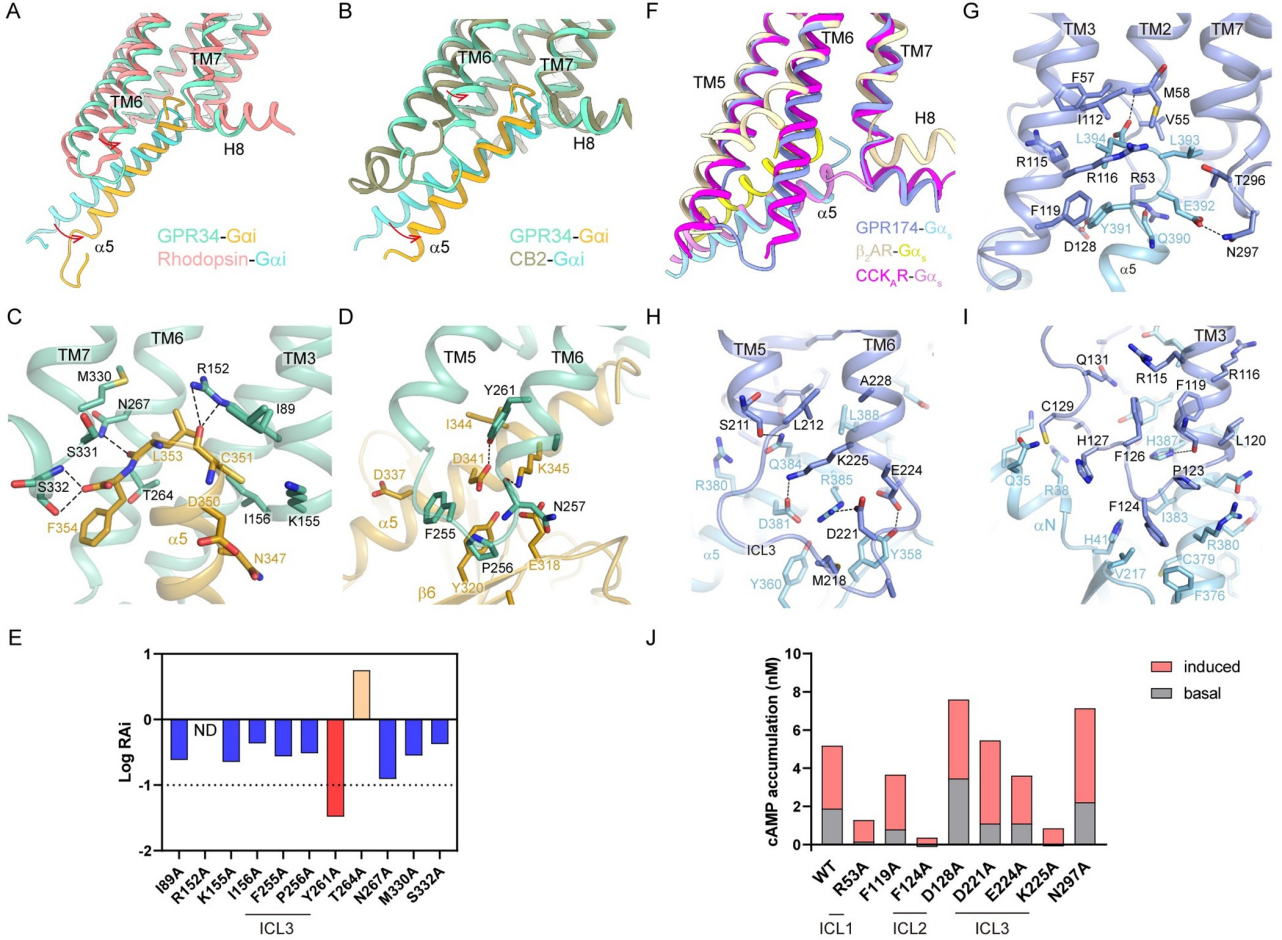
G protein coupling of GPR34 and GPR174. (**A**) Structural superposition of GPR34-Gα_i_ and rhodopsin-Gα_i_ (PDB: 6cmo). Rhodopsin is colored pink, and Gi coupled to it is colored cyan. (**B**) Structural superposition of GPR34-Gα_i_ and CB2-Gα_i_ (PDB: 6pt0). CB2 is colored brown, and Gα_i_ coupled to it is colored cyan. The displacements are indicated by arrows. (**C**) Interactions between the C terminus of α5 and GPR34. (**D**) Interactions between α5/β6 of Gα_i_ and ICL3 of GPR34. (**E**) Relative intrinsic activity (RAi) plot of GPR34 mutants of the G protein–coupling sites in Gi-dissociation assays. RAi was calculated as [E_max_(test)/EC_50_(test)]/[E_max_(WT)/EC_50_(WT)]. E_max_ and EC_50_ are from the averaged concentration-response curves of 3 independent experiments. (**F**) Superposition of GPR174-Gα_s_, β_2_AR-Gα_s_ (PDB: 3sn6), and CCK_A_R-Gα_s_ (PDB: 7ezk). GPR174, β_2_AR, and CCRK_A_R are colored blue, light yellow, and magenta, respectively. Gα_s_ subunits in these structures are colored light blue, yellow, and plum, respectively. (**G**) Interactions between the C terminus of α5 helix and GPR174. (**H**) Interactions between Gα_s_ and ICL3 of GPR174. (**I**) Interactions between Gα_s_ and ICL2 of GPR174. Polar interactions are depicted as black dashed lines. (**J**) Basal activity and maximal LysoPS-induced activity of GPR174 mutants in cAMP accumulation assays. Data are from the averaged concentration-response curves of 3 independent experiments. The data used to generate graphs in Fig 5E and 5J are available in S1 Data.

The coupling mode between GPR34 and Gi resembles that of many Gi/o-coupled class A GPCRs such as rhodopsin and cannabinoid receptor CB2. The structure of GPR34-Gi was compared with the structures of rhodopsin-Gi and CB2-Gi (Fig 5A and 5B). All of them adopt a similar conformation at the α5 helix of Gα_i_. However, TM6 of GPR34 exhibits a smaller outward displacement at the intracellular end of TM6, which results in a more upright pose of the α5 helix. TM5 is not involved in the coupling between GPR34 and Gα_i_ subunit. The C terminus of α5 inserts into a cavity mainly formed by TM3, TM6, and the linkage between TM7 and H8. Specifically, R152^3.50^, N267^6.36^, and S332^7.58^ form hydrogen bonds with main chain atoms of C351, L353, and F354 of Gα_i_, respectively (Fig 5C). In addition, I89^2.39^, K155^3.53^, I156^3.54^, T264^6.33^, M330^7.56^, and S331^7.57^ interact with α5 through hydrophobic interactions. ICL3 and the intracellular end of TM6 binds to Gα_i_ through additional interactions with α5 and β6. Specifically, N257^6.26^ and Y261^6.30^ form hydrogen bonds with K345 and D341, respectively. Hydrophobic interactions between F255 and P256 of ICL3 and I344, D337, Y320, and E318 of Gα_i_ further stabilize the interface (Fig 5D). Most mutations at the G protein–coupling sites impaired activity of GPR34 (Figs 5E, S6A and S6B). Specifically, R152A and Y261A dramatically impaired GPR34 activation.

Unexpectedly, GPR174 couples to Gs in a noncanonical manner compared with the well-studied Gs-coupled receptor β_2_AR (Fig 5F). Specifically, the displacement of TM6 in GPR174 is substantially smaller than in β_2_AR, with a distance of 12.7 Å at the Cα atom of the residue at position 6.29. Furthermore, Gα_s_ adopts a distinct conformation at the C terminus of α5 helix compared with the β_2_AR-Gs complex. Instead of turning back to interact with TM5 and TM6, the C terminus of α5 helix reaches out like a bended finger to bind into a deeper cavity formed by the TM7-H8 linkage, TM1-3, ICL1, and ICL2. Inside this pocket, F119^3.53^ of GPR174 prevents the formation of a cation–π interaction between R116^3.50^ and Y391 of Gα_s_ (S6C and S6D Fig). Y391 rotates to the direction of ICL2 and forms a hydrogen bond with D128 (Fig 5G). Cholecystokinin A receptor (CCK_A_R) was also reported to adopt a noncanonical Gs-coupling mode. It shares a similar extent of TM6 displacement with GPR174 upon Gs binding (Fig 5F). However, in CCK_A_R-Gs complex, the interaction between R^3.50^ and Y391 persists due to the absence of a phenylalanine at position 3.53 (S6E Fig), which is unique to GPR174 and P2Y10 among class A GPCRs. In the GPR174-Gs complex, the C terminus of α5 helix binds to the receptor by extensive polar interactions and hydrophobic interactions within the pocket (Fig 5G). The ICL3 loop, along with the intracellular end of TM5 and TM6, forms extensive polar interactions with residues at α5 and β6 of Gα_s_, including Y358, D381, Q384, and R385 (Fig 5H). Additionally, the ICL2 loop of GPR174 binds to the αN-α5 domain of Gα_s_ primarily through hydrophobic interactions, including the binding of F124^34.51^ in the hydrophobic pocket formed by H41, V217, F376, C379, R380, and I383 (Fig 5I), as seen in β_2_AR-Gs complex. For GPR174, some mutations at the G protein–coupling sites also impaired receptor activation (Figs 5J, S6F and S6G). Notably, F124^ICL2^A nearly abolished both spontaneous activity and LysoPS-induced activity. R53^ICL1^A and K225^ICL3^A also dramatically impaired GPR174 activation, suggesting the important roles of the intracellular loops of GPR174 in Gs coupling. Overall, the GPR174-Gs complex exhibits a unique coupling mode characterized by the lack of R^3.50^–Y391 interaction and an unusual binding pocket for the C terminus of α5 helix, which may have implications for drug discovery efforts targeting this receptor.

## Discussion

LysoPS is a lipid mediator implicated in various pathological conditions, including inflammatory and autoimmune diseases, which makes LysoPS receptors promising drug targets.

Structural analysis of GPR34 and GPR174 reveals a unique binding pocket for LysoPS, comprising an inner polar subpocket and a lateral hydrophobic subpocket. Based on structural and functional analysis, we find that GPR34 and GPR174 use different combinations of residues to recognize the polar head of LysoPS. GPR174 possesses a more positively charged pocket that accommodates the polar head of LysoPS, which may partially account for its spontaneous activity [42].

In a preprint paper [43], the authors reported 2 cryo-EM structures of GPR34-Gi complex bound with the LysoPS analogue S3E-LysoPS or its derivate M1. S3E-LysoPS is a *sn-3* LysoPS containing an ethoxy group at the *sn-1* position, while what we used in our study is *sn-1* LysoPS. M1 is a S3E-LysoPS derivate with the oleic acid substituted by an aromatic fatty acid. Similarly, they also found that the ligand-binding pocket of GPR34 is laterally open toward the membrane, and recognition of the serine moiety was realized by the charged cluster. Moreover, they found that the aromatic fatty acid surrogate of M1 forms stable hydrophobic interactions with the ligand-binding pocket, which makes M1 a super-agonist. It is interesting to see the structural basis underlying which GPR34 responds to both endogenous LysoPS and LysoPS analogues from our works. In fact, S3E-LysoPS and *sn-1* LysoPS share a similar structure, except that the *sn-1* carbon of S3E-LysoPS lies on the sidechain, shortening the backbone of S3E-LysoPS. And the carbonyl of the ester linkage is closer to R286^6.55^, with which it forms an additional hydrogen bond. The authors also found that LysoPS produced on the outer leaflet of the plasma membrane by PS-PLA1 is associated with the cells and enters the binding pocket of GPR34 laterally to activate it. This provided convincing evidence that LysoPS can enter the ligand-binding pocket from the lateral cleft between TM4 and TM5. And we think this ligand-entry mechanism may also be shared by other P2Y-like receptors.

Our structures also provide insights into the structure-activity relationships of LysoPS analogs [44,45]. From the structure, the fact that analogs with a methyl group at the β-position of the serine moiety have no activity on GPR34 may possibly result from the steric hindrance with Y135^3.33^. On the other hand, substitution of the ester linkage with an amide bond confers selectivity for GPR174, which may result from the different conformations of LysoPS molecules in the structures of GPR34 and GPR174. The ester linkage of LysoPS in GPR174 is almost identical to the *trans* configuration of an amide bond in conformation, while this is not the case in GPR34 (Fig 2). Loss of flexibility prevents formation of the optimal binding conformation and leads to reduced potency towards GPR34. Additionally, substitution of the fatty acid with specific 3-(alkoxyphenyl)propionic acid enhances potency towards LysoPS receptors possibly by forming hydrophobic and aromatic interactions with specific residues in GPR34 and GPR174. In GPR34, the benzene ring of the alkoxyphenyl group is expected to form hydrophobic interactions with M189 and F219. In GPR174, the benzene ring can also form aromatic interactions with F152 and F250.

Collectively, these results provide valuable insights into the mechanisms of ligand recognition and signaling of LysoPS receptors, as well as a structural framework for rational drug design targeting LysoPS receptors.

## Materials and methods

### Constructs

For structural studies, human GPR34 and GPR174 were codon optimized for expression in Sf9 insect cells and cloned into a modified pFastBac1 vector. At the N terminus, a bovine prolactin signal peptide was added, followed by a FLAG tag (DYKDDDD), an 8× His tag, an N-terminal fragment of β_2_ adrenergic receptor (BN, residues from 2 to 30), and a TEV site. Two copies of BN were used at the same site for GPR174. At the C terminus, an LgBiT subunit linked by a 17-amino acid linker (HMGSSGGGGSGGGGSSG) was inserted before the stop codon. Residues after Q347 and L308 were truncated in GPR34 and GPR174, respectively. An engineered Gα_i1_ protein with 4 mutations (DNGα_i_) [46] and a DN_miniGα_s_ [47] used in previous studies were also cloned into the pFastBac1 vector. Human Gβ_1_ and Gγ_2_ were cloned into the pFastBac-Dual vector with a 6× His tag at the N terminus of Gβ_1_ and a HiBiT subunit linked by a 15-amino acid linker (GSSGGGGSGGGGSSG) at the C terminus of Gβ_1_. Nb35 was cloned into the pMESy4 vector, with a pelB signal sequence at the N terminus and a 6× His tag at the C terminus. And scFv16 with an AcNPV gp67 signal peptide at the N terminus and a TEV site followed by an 8× His tag at the C terminus was cloned into the pFastBac1 vector.

For functional assays, GPR34 or GPR174 of full length was cloned into the pcDNA3.1(+) vector with an HA signal followed by a FLAG tag at the N terminus.

### Protein expression and purification

Nb35 and scFv16 were prepared as previously reported [48,49]. Briefly, Nb35 was expressed at BL21 *E*.*coli* cells and purified by His tag. scFv16 was expressed at Tni (HiFive) insect cells and purified by His tag before digested with TEV enzyme to remove His tag. Purified Nb35 and scFv16 were stored at −80°C before use.

GPR34-Gi or GPR174-Gs complex was coexpressed in Sf9 using the Bac-to-Bac system (Invitrogen). Sf9 cells at the density of 2.5 × 10^6^ cells per ml were infected with viruses for receptor, Gα, and Gβγ at the ratio of 1:1:1 and cultured at 27°C for 48 hours. Cells were collected by centrifugation and resuspended in PBS before frozen in liquid nitrogen. Cells were stored at −80°C before use.

Cells were thawed in precold lysis buffer consisting of 20 mM HEPES (pH 7.5), 50 mM NaCl, 10 mM MgCl_2_, 5 mM CaCl_2_, 25 mU/ml apyrase, 2.5 μg/ml leupeptin, 150 μg/ml benzamidine, and 100 μM TCEP and incubated at room temperature for 2 hours. Then, 18:1 LysoPS (Avanti polar lipids) was added to the lysis buffer and all the following step at the concentration of 1 μM. The sample was centrifuged at 30,700 × *g* for 30 minutes to collect cell membranes. The membranes were resuspended with a glass Dounce homogenizer in solubilization buffer consisting of 20 mM HEPES (pH 7.5), 100 mM NaCl, 1% (w/v) lauryl maltose neopentyl glycol (LMNG, Anatrace), 0.2% (w/v) cholesteryl hemisuccinate (CHS, Anatrace), 10% (v/v) glycerol, 10 mM MgCl_2_, 5 mM CaCl_2_, 12.5 mU/ml apyrase, 2.5 μg/ml leupeptin, 150 μg/ml benzamidine, and 100 μM TCEP and incubated at 4°C for 2 hours. The supernatant was isolated by centrifugation at 38,900 × g for 45 min, and then incubated with Ni resin at 4°C for 2 hours. After binding, the resin was washed with 20 column volumes of buffer A consisting of 20 mM HEPES (pH 7.5), 100 mM NaCl, 0.05% LMNG, 0.01% CHS, 2.5 μg/ml leupeptin, 150 μg/ml benzamidine, and 100 μM TCEP with 20 mM imidazole before bound protein was eluted with 5 column volumes of buffer A containing 400 mM imidazole. The eluate was supplemented with 2 mM CaCl_2_ and incubated with M1 anti-FLAG resin (Sigma Aldrich) overnight at 4°C. The resin was washed with 10 column volumes of buffer A supplemented with 2 mM CaCl_2_, and the protein was eluted with 3.5 column volumes of buffer A containing 5 mM EDTA and 200 μg/ml FLAG peptide.

Nb35 or scFv16 was added at a ratio of 1.3:1 over the complex for GPR174-Gs or GPR34-Gi, respectively. The complex was concentrated and loaded onto Superdex 200 10/300 GL column preequilibrated with running buffer containing 20 mM HEPES (pH 7.5), 100 mM NaCl, 0.00075% (w/v) LMNG, 0.00025% (w/v) glyco-diosgenin (GDN, Anatrace), 0.00015% (w/v) CHS, and 100 μM TCEP. Fractions containing monomeric complex were pooled and concentrated for electron microscopy experiments.

### Cryo-EM data acquisition

For GPR34-Gi complex, 3 μl purified protein at the concentration of 13 mg/ml was applied onto a glow-discharged holey carbon grid (Quantifoil, Au200 R1.2/1.3). For GPR174-Gs complex, 3 μl purified protein at the concentration of 3 mg/ml was applied onto a glow-discharged holey Ni-Ti alloy grid (ANTcryo, Au300 R1.2/1.3). The grids were plunge frozen in liquid ethane using Vitrobot Mark IV (Thermo Fisher Scientific). Data collection was performed on a Titan Krios electron microscope at 300 kV accelerating voltage using a Gatan K3 Summit direct electron detector with an energy filter. Micrographs were collected using the EPU software in super-resolution mode with a calibrated pixel size of 0.535 Å and a defocus range of −1.2 to −2.2 μm. Each movie was comprised of 32 frames with a total exposure dose of 55 e^−^/Å^2^.

### Cryo-EM data processing

For GPR34-Gi complex, a total of 4,381 movie stacks were subjected to Patch motion correction and Patch CTF estimation in CryoSPARC [50]. A total of 8,530,353 particles were picked by Template picker and then subjected to 2D classification to discard poorly defined particles. After 3 rounds of 3D classification with Ab-initio reconstruction and Heterogeneous refinement, a subset with 304,493 particles were selected for Non-Uniform refinement [51]. The particles were imported into Relion [52] and classified by 3D classification without alignment. The classification was focused on the receptor by a mask. The best class was selected for Non-Uniform refinement, which generated a map with an indicated global resolution of 2.91 Å at a Fourier shell correlation (FSC) of 0.143 (S2 Fig).

For GPR174-Gs complex, 2,043 movies were imported into CryoSPARC [50] and preprocessed by Patch motion correction and Patch CTF correction. Approximately 1,486 micrographs with reasonable total motion distance and CTF resolution were selected for further processing. 2D templates for particle picking were created by Blob picker and 2D classification using 100 micrographs. A total of 2,561,330 particles were picked by Template piker from all micrographs. The particles were subjected to 2D classification, and 533,772 particles were selected for 3D reconstruction. Particles were classified by Heterogeneous Refinement using templates generated by Ab-initio Reconstruction. Then, 329,915 particles from the best 2 classes were selected for another round of 3D classification. Finally, 132,808 particles were selected and processed by Non-uniform Refinement [51]. The map was further improved by Local Refinement with a global mask, resulting in a map at resolution of 3.06 Å estimated by gold-standard FSC at the threshold of 0.143 (S3 Fig). Local resolution was estimated in CryoSPARC. And the final map was locally filtered based on local resolution for model building and visualization.

### Model building and refinement

Structures of GPR34 and GPR174 predicted by AlphaFold [53,54] were used as initial models for the receptors. The coordinates of DNGi and scFv16 were derived from the structure of D2R-Gi-scFv16 complex (PDB: 7JVR) [47], and the coordinates of miniGs and Nb35 were derived from the structure of D1R-Gs-Nb35 complex (PDB: 7JVQ) [47]. Different subunits were docked into the density maps in UCSF Chimera. Then, the initial models were manually adjusted in Coot [55] and autorefined by Phenix [56]. Ligands were generated by the eLBOW program in Phenix. The model statistics were calculated by MolProbity [57] (S1 Table).

### G protein-dissociation assay

The function of GPR34 and its mutants was analyzed by a G protein-dissociation assay based on the TRUPATH platform [58]. HEK293T cells were transfected with wild-type or mutant GPR34, Gα_i1_-Rluc8, Gβ_3_, Gγ_9_-GFP2 plasmid at the ratio of 1:1:1:1 in 6-cm dishes using lipofectamine 3000 (Thermo Fisher Scientific). About 40 hours after transfection, cells were collected by trypsin-EDTA treatment and resuspended in BRET buffer (1× HBSS, 25 mM HEPES (pH 7.4), 0.1% BSA). Cells were plated in 96-well white wall, white-bottom plates at the density of 100,000 cells in 30 μl per well. Then, 30 μl substrate solution with 15 μM coelenterazine 400a (Nanolight Technology) in BRET buffer was added to each well, and the plate was incubated at room temperature for 5 minutes. Finally, 30 μl 3× LysoPS (18:1 or 18:0) diluted in BRET buffer at different concentrations was added to each well. The plate was incubated at room temperature for 5 minutes before tested on a SpectraMax iD5 (Molecular Devices) microplate reader. BRET signal was measured using 515 nm and 410 nm emission filters. BRET ratio (515 nm/410 nm) was normalized to the ligand-free control before further analysis. Outcomes of 3 independent assays (each in triplicate) were used to calculate a concentration-response curve with a 3-parameter logistic function. All curves were further aligned by the “Bottom” parameter.

### cAMP accumulation assay

The function of GPR174 and its mutants was analyzed by cAMP accumulation assay with a cAMP HTRF kit (Cisbio). CHO-K1 cells were transfected with wild-type or mutant GPR174 plasmids in 12-well plates using lipofectamine 3000 (Thermo Fisher Scientific). About 44 hours after transfection, cells were collected by trypsin-EDTA treatment and resuspended in Stimulation buffer supplemented with 0.5 mM IBMX. Cells were plated in 384-well plates at the density of 2,500 cells in 5 μl per well. And 5 μl 2× LysoPS (18:1) diluted in Stimulation buffer with 0.5 mM IBMX at different concentrations was added to each well. The plate was incubated at 37°C for 30 minutes. Then, 10 μl detection solution with Eu-cAMP and d2-antibody was added to each well. Then, the plate was incubated at room temperature for 1 hour before tested on a SpectraMax iD5 (Molecular Devices) microplate reader. FRET signal was measured at the excitation wavelength of 340 nm and the emission wavelength of 665 nm and 616 nm using filters. Outcome of 3 independent assays (each in duplicate) was used for analysis. The HTRF ratio (665 nm/616 nm) was transformed into the concentration of cAMP by a standard curve. The concentration-response curve was calculated with a 3-parameter logistic function.

### Expression analysis of mutants

The expression of GPR34 or GPR174 and their mutants was analyzed by flow cytometry. In consistence with the functional assays, HEK293T and CHO-K1 cells were used for GPR34 and GPR174, respectively. Cells were transfected with wild-type or mutant receptor plasmid in 24-well plates using lipofectamine 3000 (Thermo Fisher Scientific). After 2 days, cells were collected by trypsin-EDTA treatment and resuspended in HBSS. Cells at a density of 40,000 to 60,000 cells in 20 μl was mixed with 20 μl anti-FLAG M2-FITC antibody (Sigma Aldrich, for GPR34) or Alexa Fluor 647 anti-FLAG antibody (Abcam, for GPR174) diluted in TBS buffer with 4% BSA. The sample was incubated at 4°C for 20 minutes before 160 μl HBSS with 5 mM HEPES (pH 7.4) was added. Fluorescence signal was measured on CytoFLEX (Beckman), and 10,000 cells were recorded for each sample. Single cells were defined by setting FSC/SSC thresholds, and the median fluorescence intensity was used to evaluate receptor expression. Data were normalized to the expression level of wild-type receptor (100%) and mock-transfected cells (0%). Outcomes of 3 independent assays (each in duplicate) were used for analysis.

## Supporting information

S1 Fig Lysophospholipids and lysophospholipid receptors. (**A**) Lysophospholipids. (**B**) Phylogenetic tree of lysophospholipid receptors. Sequence similarity analysis of EDG family receptors, P2Y family receptors, and other lysophospholipid receptors. Multiple sequence alignment was done with MUSCLE. Phylogenetic tree was calculated by neighbor-joining method and displayed by iTOL. EDG family and P2Y family are colored red and green, respectively.
(TIF)

S2 Fig GPR34-Gi complex preparation and cryo-EM data processing. (**A**) Cryo-EM image processing workflow for GPR34-Gi complex. (**B**) SDS-PAGE profile of GPR34-Gi-scFv16 complex. Uncropped gel for S2B is provided in S1 Raw Images. (**C**) Representative cryo-EM image (scale bar: 50 nm). (**D**) Representative 2D class averages (scale bar: 5 nm). (**E**) Angular distribution plot of final particles. (**F**) The “gold-standard” FSC curves of the GPR34-Gi-scFv16 complex. (**G**) Cryo-EM density maps and models of the 7 transmembrane helices (TM1-7), Helix 8 (H8), α5 helix of Gα_i_, and the ligand of LysoPS 18:1 bound GPR34-Gi complex are shown. The EM density is shown at the threshold of 0.3.
(TIF)

S3 Fig GPR174-Gs complex preparation and cryo-EM data processing. (**A** and **B**) Size-exclusion chromatography and SDS-PAGE profiles of GPR174-Gs-Nb35 complex. Uncropped gel for S3B is provided in S1 Raw Images. (**C**) Representative cryo-EM image (scale bar: 50 nm). (**D**) Flow chart of cryo-EM data processing and local resolution of the final map. Density of the ligand in the final map is indicated by a black dashed ellipse. (**E**) Representative 2D class averages (scale bar: 5 nm). (**F**) Angular distribution plot of final particles. (**G**) The “gold-standard” FSC curves of the GPR174-Gs-Nb35 complex. (**H**) Density maps of the 7 transmembrane helices (TM1-7), Helix 8 (H8), α5 helix of Gα_s_ and LysoPS bound in GPR174-Gs complex are shown. The EM density is shown at the threshold of 0.276.
(TIF)

S4 Fig Functional data of GPR34. (**A**) Activity of GPR34 induced by 18:1 or 18:0 LysoPS in the Gi-dissociation assay. (**B**) Expression of GPR34 mutants in HEK293T cells. (**C**-**E**) Concentration-response curves of GPR34 mutants in the Gi-dissociation assay. Data represent mean ± SEM from at least 3 independent experiments. The data used to generate graphs in S4A-S4E are available in S1 Data.
(TIF)

S5 Fig Functional data of GPR174. (**A**) Expression of GPR174 mutants in CHO cells. (**B**-**D**) Concentration-response curves of GPR174 mutants in the cAMP accumulation assay. Data represent mean ± SEM from 3 independent experiments. The data used to generate graphs in S5A-S5D are available in S1 Data.
(TIF)

S6 Fig Functional data of G protein coupling. (**A**) Concentration-response curves of GPR34 mutants in the Gi-dissociation assay. (**B**) Expression of GPR34 mutants in HEK293T cells. (**C**-**E**) Details of Gα_s_ binding by GPR174 (**C**), β_2_AR (**D**, PDB: 3sn6), and CCK_A_R (**E**, PDB: 7ezk). (**F**) Concentration-response curves of GPR174 mutants in the cAMP accumulation assay. (**G**) Expression of GPR174 mutants in CHO cells. All data represent mean ± SEM from at least 3 independent experiments. The data used to generate graphs in S6A, S6B, S6F, and S6G are available in S1 Data.
(TIF)

S1 Table Cryo-EM data collection, model refinement, and validation statistics. (DOCX)

S1 Data Data used for graphs in Figs 2B, 2D, 5E, 5J, S4A–S4E, S5A–S5D, S6A, S6B, S6F and S6G. (XLSX)

S1 Raw Images Uncropped Coomassie-stained SDS-PAGE gels used for S2B and S3B Figs. (PDF)

## Abbreviations

CCK_A_R: cholecystokinin A receptor
CHS: cholesteryl hemisuccinate
EDG: endothelial differentiation gene
FSC: Fourier shell correlation
LMNG: lauryl maltose neopentyl glycol
LPA: lysophosphatidic acid
LPC: lysophosphatidylcholine
LPL: lysophospholipid
LysoPS: lysophosphatidylserine
PAFR: platelet activating factor receptor
S1P: sphingosine 1-phosphate
TGFα: transforming growth factor-α
TMD: transmembrane domain
Treg: regulatory T cell
