# Supplementary figures and images for: Structural basis for ligand recognition and signaling of the lysophosphatidylserine receptors GPR34 and GPR174
Source: PLoS Biol. 2023 Dec 4;21(12):e3002387. doi: 10.1371/journal.pbio.3002387 (PMC10721165; doi:10.1371/journal.pbio.3002387)

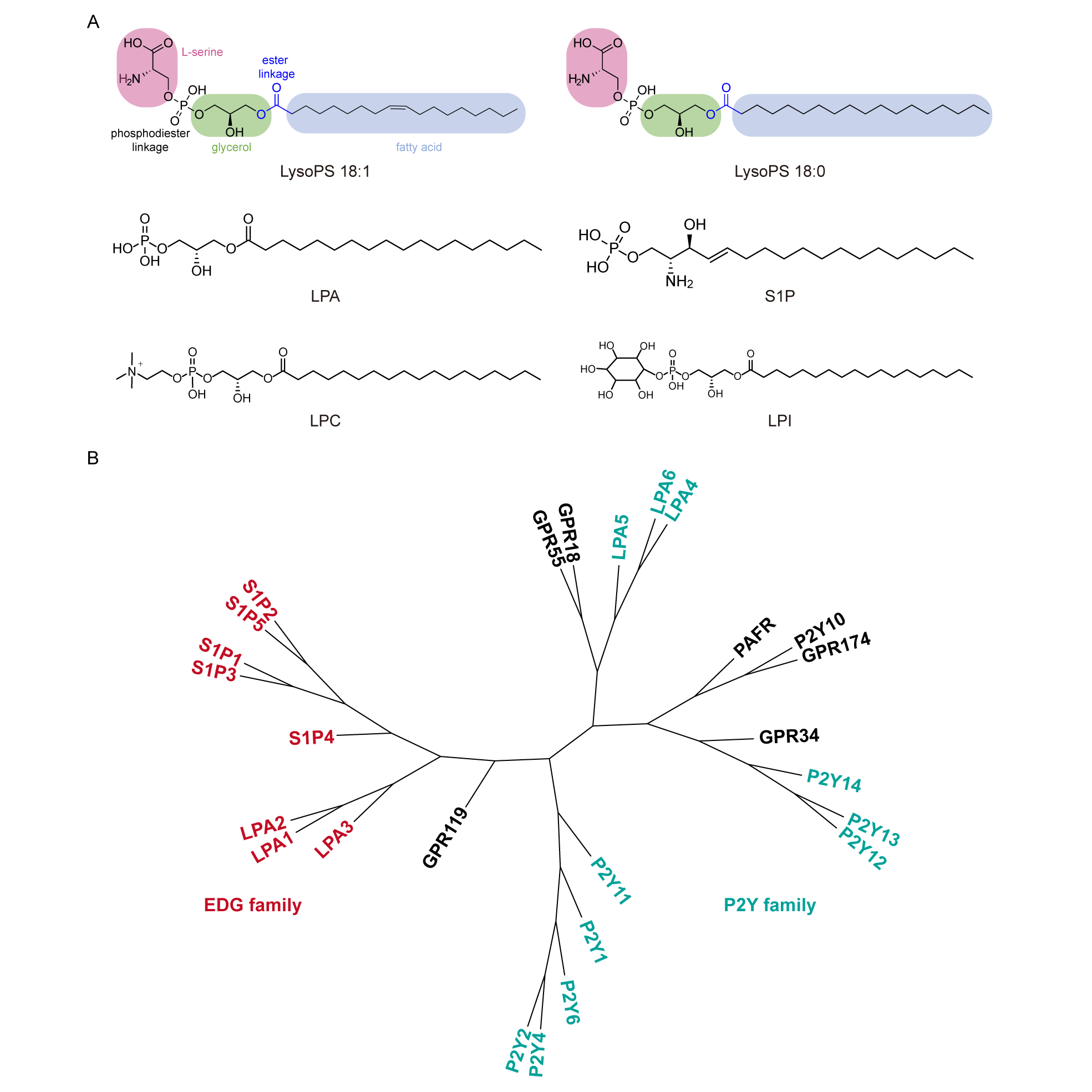

Supplement: S1 Fig — (A) Lysophospholipids. (B) Phylogenetic tree of lysophospholipid receptors. Sequence similarity analysis of EDG family receptors, P2Y family receptors, and other lysophospholipid receptors. Multiple sequence alignment was done with MUSCLE. Phylogenetic tree was calculated by neighbor-joining method and displayed by iTOL. EDG family and P2Y family are colored red and green, respectively. (TIF) [file pbio.3002387.s001.tif]

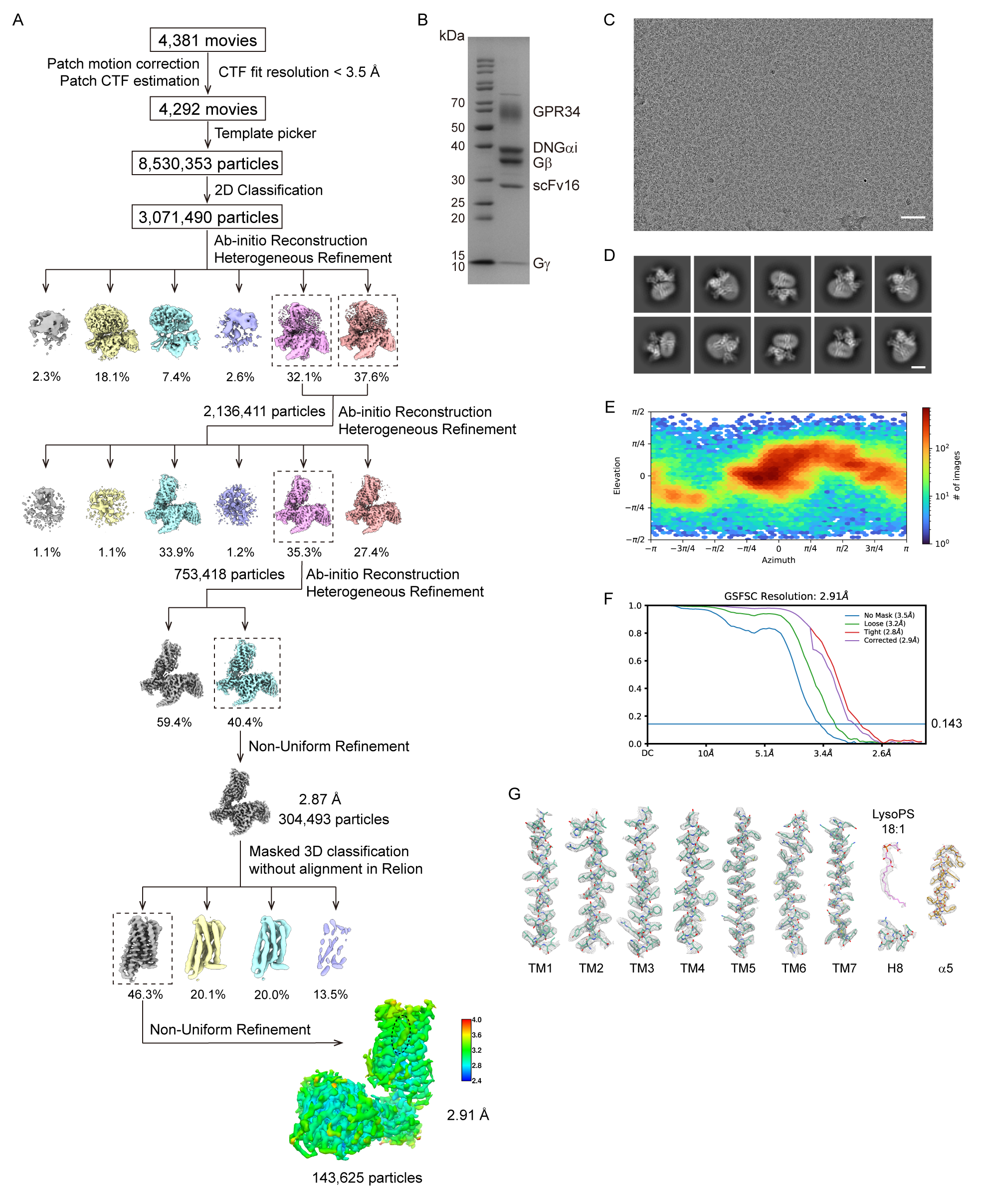

Supplement: S2 Fig — (A) Cryo-EM image processing workflow for GPR34-Gi complex. (B) SDS-PAGE profile of GPR34-Gi-scFv16 complex. Uncropped gel for S2B is provided in S1 Raw Images. (C) Representative cryo-EM image (scale bar: 50 nm). (D) Representative 2D class averages (scale bar: 5 nm). (E) Angular distribution plot of final particles. (F) The “gold-standard” FSC curves of the GPR34-Gi-scFv16 complex. (G) Cryo-EM density maps and models of the 7 transmembrane helices (TM1-7), Helix 8 (H8), α5 helix of Gαi, and the ligand of LysoPS 18:1 bound GPR34-Gi complex are shown. The EM density is shown at the threshold of 0.3. (TIF) [file pbio.3002387.s002.tif]

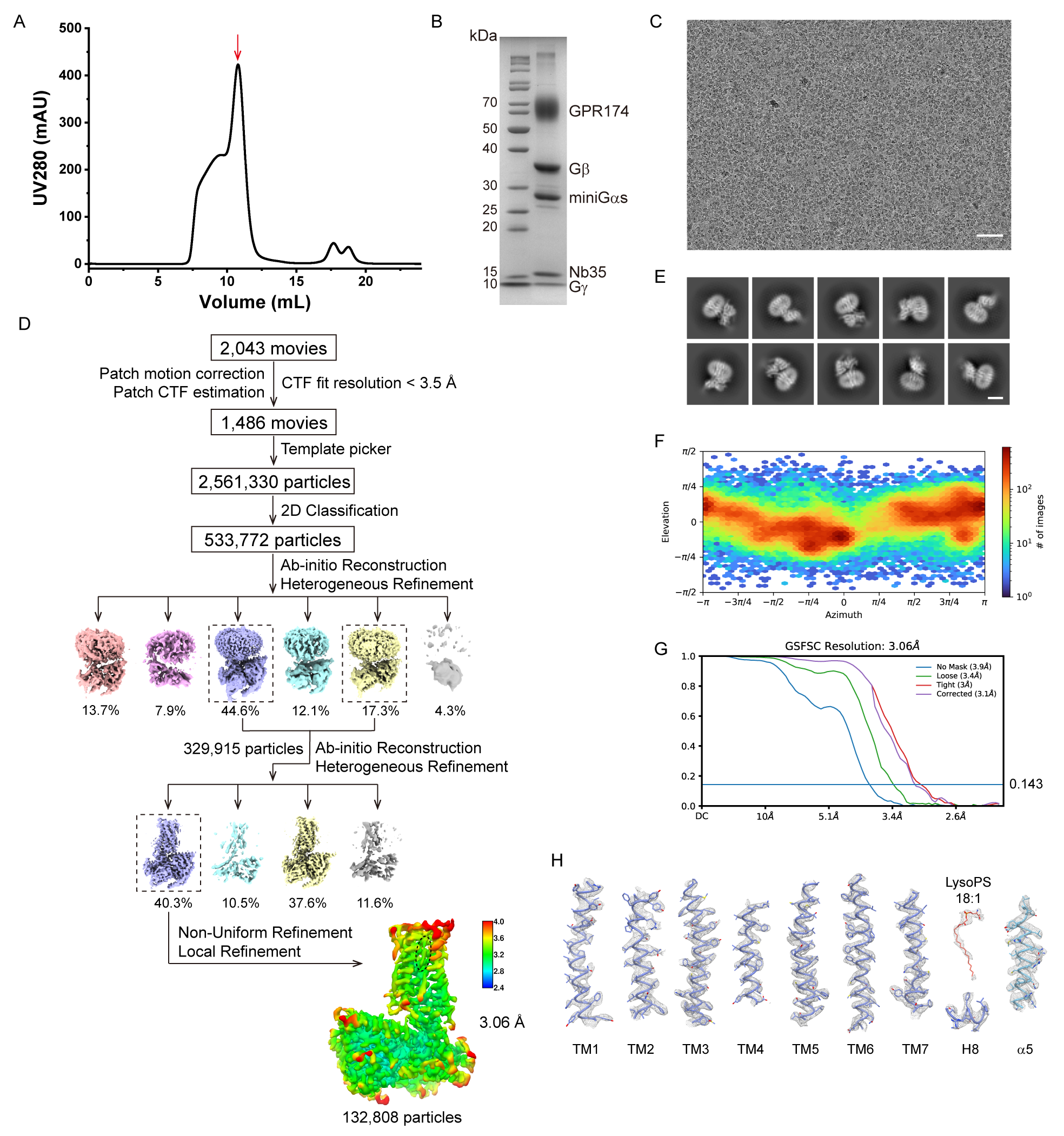

Supplement: S3 Fig — (A and B) Size-exclusion chromatography and SDS-PAGE profiles of GPR174-Gs-Nb35 complex. Uncropped gel for S3B is provided in S1 Raw Images. (C) Representative cryo-EM image (scale bar: 50 nm). (D) Flow chart of cryo-EM data processing and local resolution of the final map. Density of the ligand in the final map is indicated by a black dashed ellipse. (E) Representative 2D class averages (scale bar: 5 nm). (F) Angular distribution plot of final particles. (G) The “gold-standard” FSC curves of the GPR174-Gs-Nb35 complex. (H) Density maps of the 7 transmembrane helices (TM1-7), Helix 8 (H8), α5 helix of Gαs and LysoPS bound in GPR174-Gs complex are shown. The EM density is shown at the threshold of 0.276. (TIF) [file pbio.3002387.s003.tif]

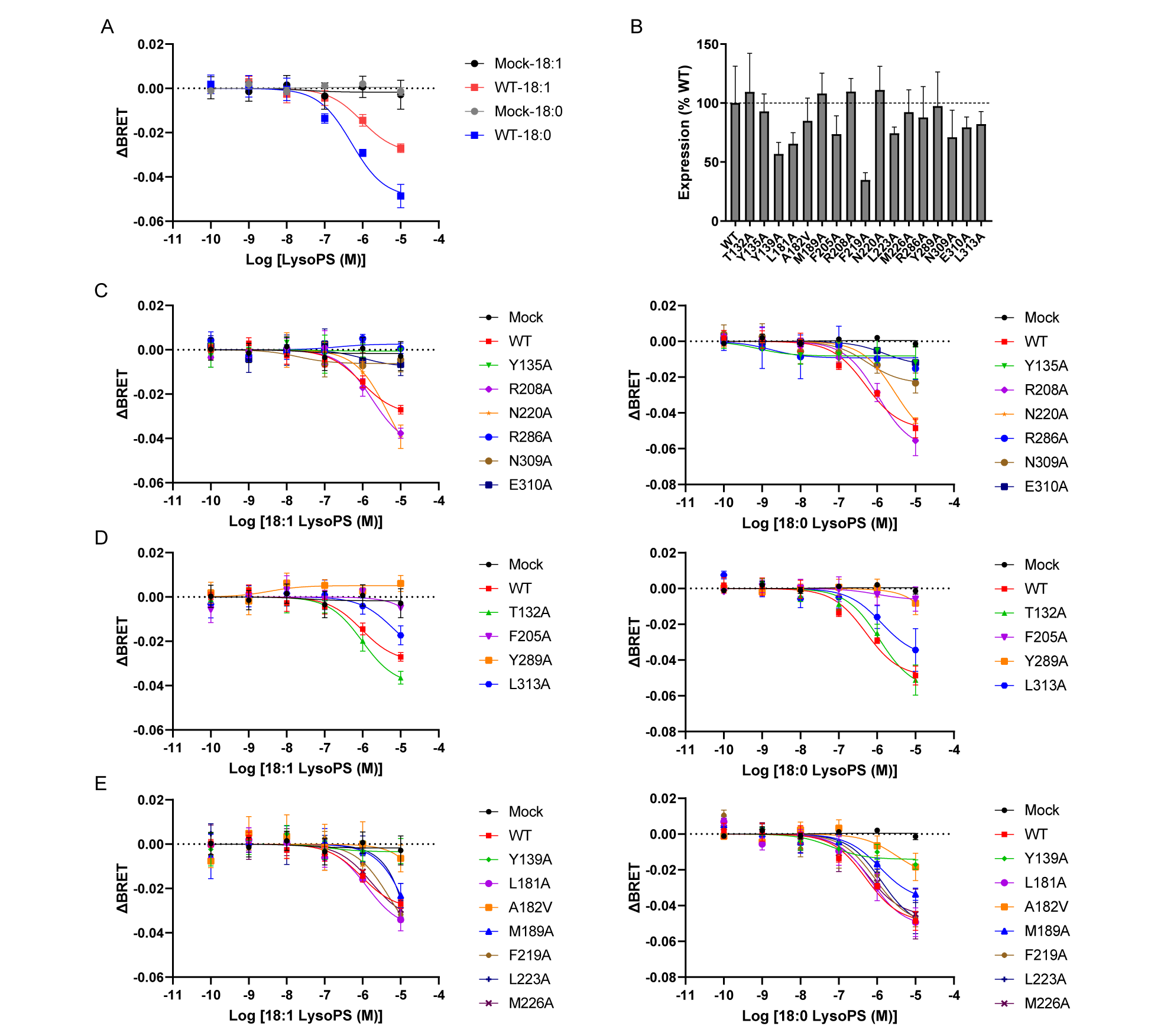

Supplement: S4 Fig — (A) Activity of GPR34 induced by 18:1 or 18:0 LysoPS in the Gi-dissociation assay. (B) Expression of GPR34 mutants in HEK293T cells. (C-E) Concentration-response curves of GPR34 mutants in the Gi-dissociation assay. Data represent mean ± SEM from at least 3 independent experiments. The data used to generate graphs in S4A-S4E are available in S1 Data. (TIF) [file pbio.3002387.s004.tif]

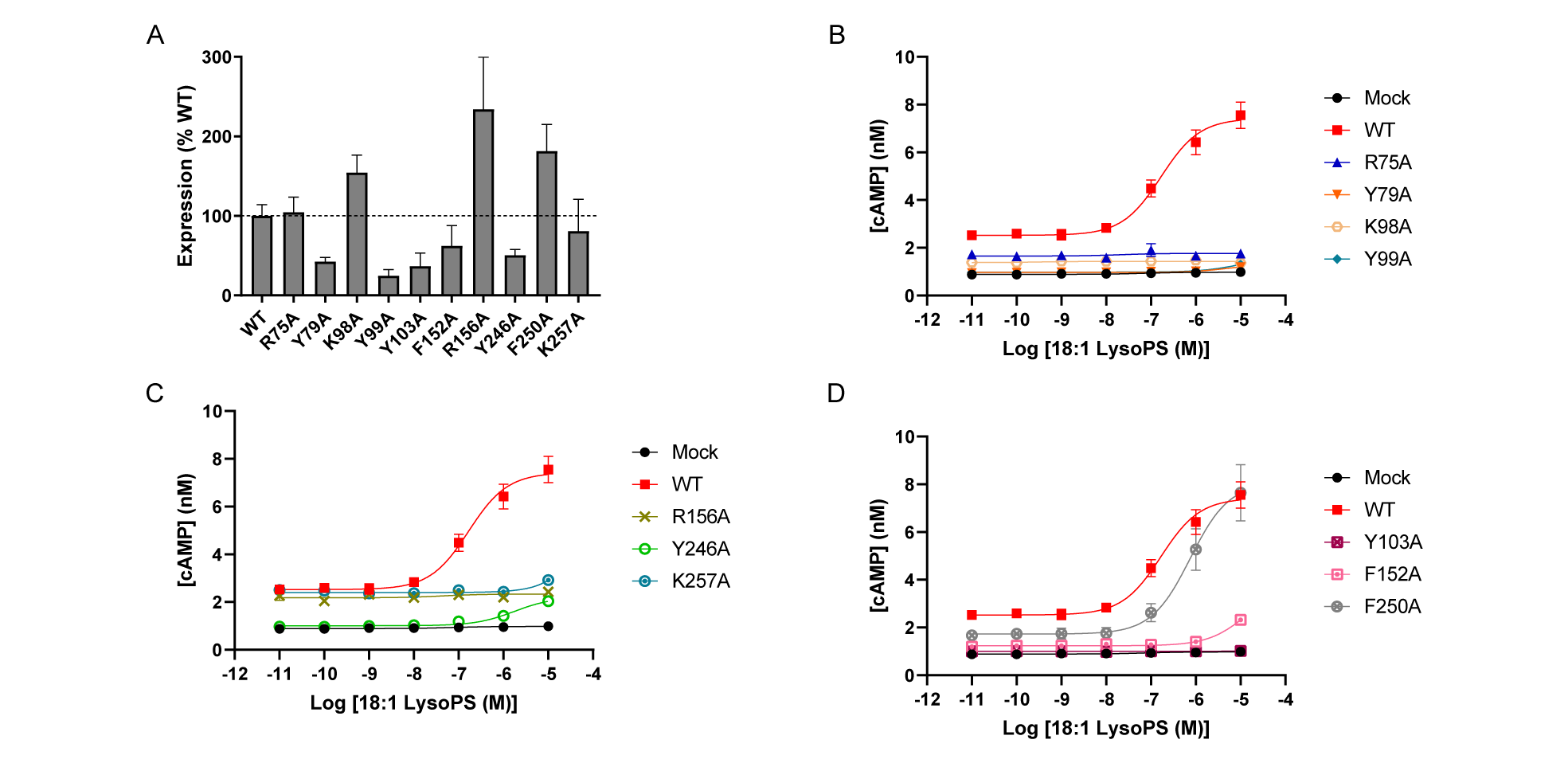

Supplement: S5 Fig — (A) Expression of GPR174 mutants in CHO cells. (B-D) Concentration-response curves of GPR174 mutants in the cAMP accumulation assay. Data represent mean ± SEM from 3 independent experiments. The data used to generate graphs in S5A-S5D are available in S1 Data. (TIF) [file pbio.3002387.s005.tif]

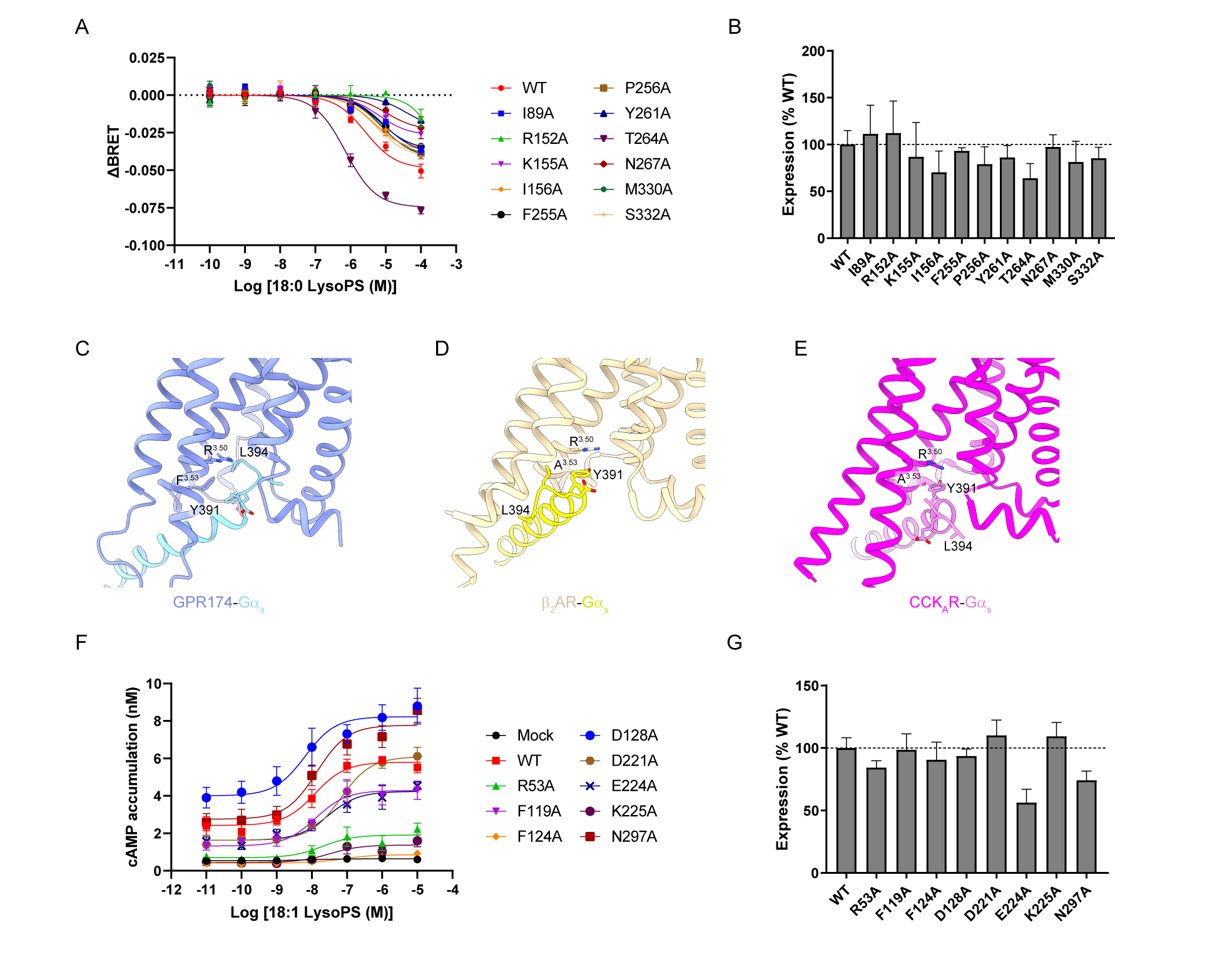

Supplement: S6 Fig — (A) Concentration-response curves of GPR34 mutants in the Gi-dissociation assay. (B) Expression of GPR34 mutants in HEK293T cells. (C-E) Details of Gαs binding by GPR174 (C), β2AR (D, PDB: 3sn6), and CCKAR (E, PDB: 7ezk). (F) Concentration-response curves of GPR174 mutants in the cAMP accumulation assay. (G) Expression of GPR174 mutants in CHO cells. All data represent mean ± SEM from at least 3 independent experiments. The data used to generate graphs in S6A, S6B, S6F, and S6G are available in S1 Data. (TIF) [file pbio.3002387.s006.tif]

**Figure S2B**

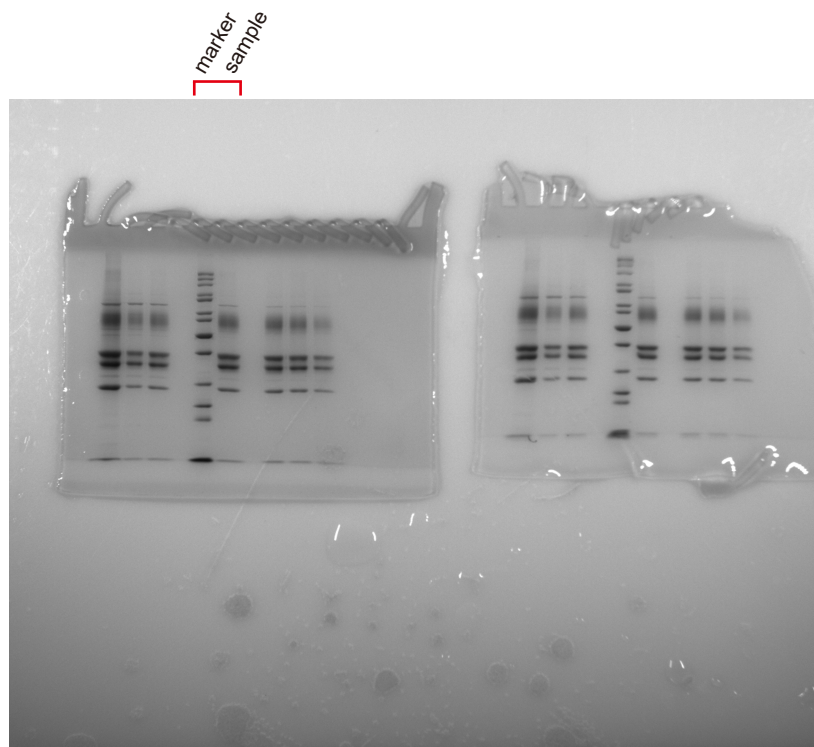

**Figure S3B**

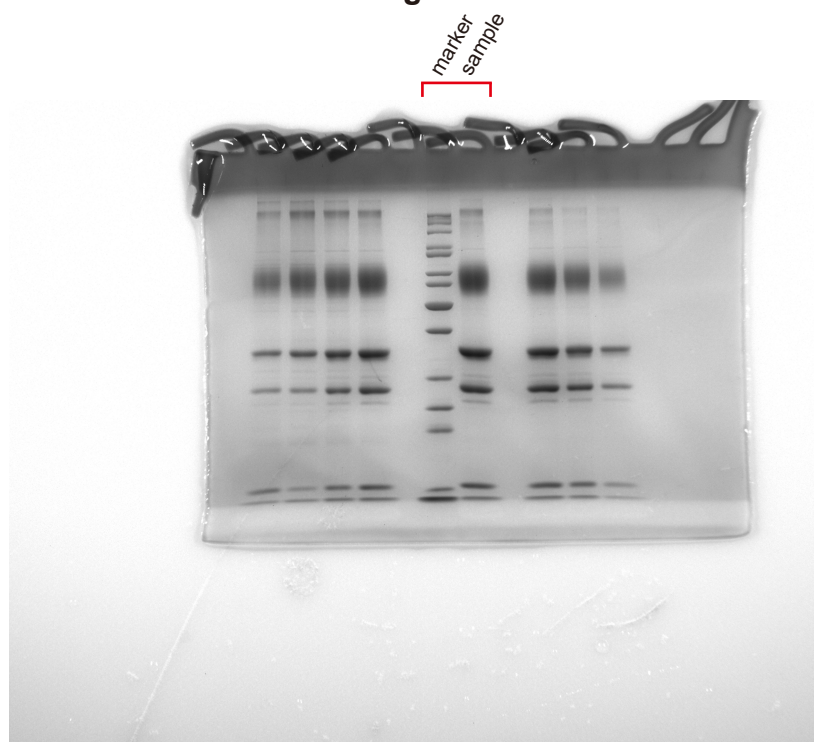

Supplement: S1 Raw Images — (PDF) [file pbio.3002387.s009.pdf]
