# Supplementary material for: Structural basis for ligand recognition and signaling of the lysophosphatidylserine receptors GPR34 and GPR174
Source: PLoS Biol. 2023 Dec 4;21(12):e3002387. doi: 10.1371/journal.pbio.3002387 (PMC10721165; doi:10.1371/journal.pbio.3002387)
Supplement: S1 Table — (DOCX) [file pbio.3002387.s007.docx]

## S1 Table. Cryo-EM data collection, model refinement and validation statistics.

|  | **GPR34-Gi**  **LysoPS (18:1)**  **(EMD-37771)**  **(PDB 8WRB)** | **GPR174-Gs**  **LysoPS (18:1)**  **(EMD-35838)**  **(PDB 8IZB)** |
| --- | --- | --- |
| **Data collection and processing** |  |  |
| Magnification | 81,000 × | 81,000 × |
| Voltage (kV) | 300 | 300 |
| Electron exposure (e^-^/Å^2^) | 55 | 55 |
| Defocus range (μm) | -2.2~-1.2 | -2.2~-1.2 |
| Pixel size (Å) | 1.07 | 1.07 |
| Symmetry imposed | C1 | C1 |
| Initial particle images (no.) | 8,530,353 | 2,561,330 |
| Final particle images (no.) | 143,625 | 132,808 |
| Map resolution (Å) | 2.91 | 3.06 |
| FSC threshold | 0.143 | 0.143 |
| **Refinement** |  |  |
| Model resolution (Å) | 3.10 | 3.20 |
| FSC threshold | 0.5 | 0.5 |
| Model composition |  |  |
| Non-hydrogen atoms | 8842 | 8322 |
| Protein residues | 1134 | 1045 |
| Ligands | 2 | 3 |
| B factors (Å^2^) |  |  |
| Protein | 84.26 | 74.46 |
| Ligand | 75.55 | 77.05 |
| R.m.s. deviations |  |  |
| Bond lengths (Å) | 0.001 | 0.001 |
| Bond angles (°) | 0.346 | 0.364 |
| **Validation** |  |  |
| MolProbity score | 1.19 | 1.27 |
| Clash score | 4.01 | 5.10 |
| Rotamer outliers (%) | 0.95 | 0.68 |
| Ramachandran plot |  |  |
| Favored (%) | 98.57 | 98.45 |
| Allowed (%) | 1.43 | 1.55 |
| Outliers (%) | 0 | 0 |
